# Molecular subtyping and improved treatment of neurodevelopmental disease

**DOI:** 10.1186/s13073-016-0278-z

**Published:** 2016-02-25

**Authors:** Holly A. F. Stessman, Tychele N. Turner, Evan E. Eichler

**Affiliations:** Department of Genome Sciences, University of Washington School of Medicine, Seattle, WA 98195 USA; Howard Hughes Medical Institute, Seattle, WA 98195 USA

## Abstract

The next-generation sequencing revolution has substantially increased our understanding of the mutated genes that underlie complex neurodevelopmental disease. Exome sequencing has enabled us to estimate the number of genes involved in the etiology of neurodevelopmental disease, whereas targeted sequencing approaches have provided the means for quick and cost-effective sequencing of thousands of patient samples to assess the significance of individual genes. By leveraging such technologies and clinical exome sequencing, a genotype-first approach has emerged in which patients with a common genotype are first identified and then clinically reassessed as a group. This approach has proven a powerful methodology for refining disease subtypes. We propose that the molecular characterization of these genetic subtypes has important implications for diagnostics and also for future drug development. Classifying patients into subgroups with a common genetic etiology and applying treatments tailored to the specific molecular defect they carry is likely to improve management of neurodevelopmental disease in the future.

## A shift to a genotype-first approach

Neurodevelopmental disorders (NDs) refer to a complex collection of phenotypes that encompass clinically recognizable disorders such as autism spectrum disorders (ASD), intellectual disability (ID), epilepsy and schizophrenia. The diagnosis of NDs has classically fallen within the clinical realm. The diagnosis of epilepsy is somewhat quantitative, with the frequency, onset and family history of seizure events being considered for classification [1], whereas the diagnosis of ASD, ID and schizophrenia is historically more complex. The Diagnostic and Statistical Manual of Mental Disorders (DSM, currently DSM-5) is recognized by the US healthcare system as a standard battery of diagnostic criteria for classifying mental disorders. These criteria recognize patients with ASD as those with primarily communication deficits, which can be measured by several standardized tests (e.g., ADOS, ADI-R and BAPQ). In addition to intelligence quotient (IQ) testing, ID is classified by the DSM-5 as involving adaptive functioning impairments in the conceptual, social and practical skills domains. Individuals diagnosed with schizophrenia must present with at least two disease-associated symptoms, which include delusions, hallucinations, disorganized speech and behavior, and social/occupational dysfunction [2].

Earlier versions of the DSM included phenotypic subtypes for many mental health disorders that have since been eliminated owing to inconsistent diagnoses between clinicians. However, the study of these disorders, ASD and ID in particular, has shown that disease subtypes do exist (such as high-functioning ASD, previously Asperger syndrome) [3]. Twin studies of ASD, epilepsy and schizophrenia showed that NDs have a strong genetic component (heritability [h^2^] = 40–80 % [4–6], h^2^ = 70–88 % [7], and h^2^ = 64–81 % [8, 9], respectively). The existence of extensive comorbidity among ND diagnoses has long been recognized; for example, 28 % of individuals who have ID also present with ASD [10], whereas 26 % present with epilepsy [11] and 3.7–5.2 % with schizophrenia [12]. Phenotypic overlap between NDs led to an early hypothesis that common risk genes underlie multiple NDs and, furthermore, that genetic characterization could be a useful diagnostic tool for ND identification and treatment [13].

Studies of copy number variation and whole-exome or whole-genome sequencing (WES and WGS, respectively) of families have highlighted the importance of rare, de novo gene-disruptive mutations in the genetic etiology of NDs. These studies frequently implicated the same copy number variant, biochemical pathway or even the same gene as an underlying factor of seemingly diverse clinical and etiological outcomes (Table 1). One classic example of this genetic overlap is a microdeletion in chromosome 15q11.3, which has been associated with multiple NDs (ASD, ID, epilepsy and schizophrenia) [14]. At the single-gene level, exome sequencing studies have highlighted that specific loci, such as *SYNGAP1*, *ARID1B* and *ADNP*, are likely to contribute to both ASD and ID, whereas mutations in genes such as *STXBP1* and *WDR45* might contribute to ID and epilepsy but not ASD (Table 1). Recognition of this genetic overlap and the subtlety of the clinical diagnoses of NDs have led to the development of a so-called genotype-first approach, in which patients with a common genotype (i.e., a disruptive variation in the same gene) are collected for deep clinical phenotyping to define the specific disease attributes associated with each candidate ND risk gene [15]. This approach contrasts with phenotype-driven approaches, in which patients are collected on the basis of a shared clinical presentation and used to identify candidate risk genes post hoc.

**Table 1:** Top de novo genes associated with ND risk

Gene	ASD^b^	ID/DD^c^	EP^d^	SZ^e^	Total cases	Control counts^f^	*p* value	OR (95 % CI)	Pathway^g^
*SYNGAP1* ^a^	7	9	0	0	16	1	1.45E-12	88.0 (13.7–3613.0)	S
*SCN2A* ^a^	6	7	0	1	14	1	1.34E-11	86.7 (13.2–3587.4)	S
*ARID1B* ^a^	5	11	0	0	16	5	9.29E-10	17.6 (6.2–61.5)	C, T, W
*ANKRD11* ^a^	2	8	0	0	10	1	2.63E-08	61.9 (8.8–2644.0)	D
*CHD8* ^a^	12	0	0	1	13	4	3.52E-08	17.9 (5.5–75.2)	C, T, W
*ADNP* ^a^	5	4	0	0	9	2	1.99E-06	24.7 (5.1–234.2)	T
*DYRK1A* ^a^	5	3	0	0	8	1	2.46E-06	44.0 (5.9–1929.7)	K, D
*CTNNB1* ^a^	1	6	0	0	7	1	1.42E-05	38.5 (4.9–1716.8)	T, W
*CHD2* ^a^	5	2	1	0	8	3	3.36E-05	14.7 (3.5–85.7)	C, T
*STXBP1*	0	5	1	0	6	1	8.12E-05	32.9 (4.0–1503.0)	S
*POGZ*	3	2	0	1	6	2	1.58E-04	18.5 (3.3–187.6)	R
*MED13L*	2	4	0	0	6	2	2.82E-04	16.5 (2.9–166.8)	T, W
*TRIP12*	2	2	0	0	4	0	5.63E-04	∞ (3.6–∞)	U
*KMT2A*	2	4	0	0	6	4	9.24E-04	9.3 (2.2–44.7)	C
*EP300*	1	4	0	0	5	3	2.03E-03	10.3 (2.0–66.5)	C, T, W
*GRIN2B*	3	1	0	0	4	1	2.47E-03	22.0 (2.2-1075.1)	S
*DDX3X* ^a^	1	2	0	0	3	0	2.70E-03	∞ (2.6–∞)	T, W
*SUV420H1*	3	0	0	0	3	0	2.70E-03	∞ (2.6–∞)	C, T
*WDR45*	0	2	1	0	3	0	2.70E-03	∞ (2.6–∞)	A
*CHAMP1* ^a^	0	3	0	0	3	0	3.66E-03	∞ (2.3–∞)	R
*SCN1A* ^a^	0	0	4	0	4	2	6.50E-03	11.0 (1.6–121.5)	S
*WAC* ^a^	2	2	0	0	4	2	6.50E-03	11.0 (1.6–121.5)	C, T
*AHDC1* ^a^	1	2	0	0	3	1	9.68E-03	18.5 (1.5–967.5)	Db
*SATB2*	0	3	0	0	3	1	9.68E-03	18.5 (1.5–967.5)	C, T

All counts represent de novo mutations that are likely to be gene-disruptive, including frameshift, splice and nonsense mutations ^a^Gene also identified through genotype-first approaches. ^b^5001–5922 individuals with ASD were screened depending on the gene. ASD data have been previously published [19, 23–28]. ^c^1284 individuals with ID/DD were screened. ID/DD data have been previously published [29–31]. ^d^274 individuals with EP were screened. EP data have been previously published [32, 33]. ^e^785 individuals with SZ were screened. SZ data have been previously published [34–36]. ^f^Data from 45,376 control individuals were obtained from the ExAC database. The disruptive mutations counted here represent unaffected population control individuals and individuals with diseases other than neuropsychiatric disorders [37]. These data were used to calculate the Fisher’s exact test *p* value. Only disruptive (frameshift, splice, nonsense) variants were scored in cases and controls. ^g^Pathway annotations determined using the Database for Annotation, Visualization and Integrated Discovery (DAVID) v6.7 [57, 58]. *A* Autophagy, *ASD* Autism spectrum disorders, *C* Chromatin remodeling, *CI* Confidence interval, *D* Broad development, *Db* DNA binding, *DD* Developmental delay, *EP* Epilepsy, *ID* Intellectual disability, *K* Kinase, *OR* Odds ratio, *R* Replication, *S* Synapse function, *SZ* Schizophrenia, *T* Gene transcription, *U* E3 ubiquitin-protein ligase, *W* Wnt/β-catenin signaling

The goal of this Opinion is to review advances in the discovery of candidate genes based on next-generation sequencing of patients and the impact of these advances on refining specific subtypes of ND. Linking genotypes to deep clinical phenotypes (including information obtained through application of best-practice DSM-5 criteria, clinical dysmorphology assessment, analysis of family histories and electroencephalography) is providing important insight into ND risk gene models [16–18]. We propose that grouping patients on the basis of a shared genetic etiology is a critical first step in tailoring improved therapeutics to a defined subset of patients.

## Gene discovery and phenotypic refinement

The affordable application of next-generation sequencing in the clinical and research arenas has rapidly increased our understanding of the genetic variation that underlies NDs. Exome and targeted sequencing studies of patients with ND have revealed dozens of new genes emerging as high-risk candidate loci in recent years (Table 2). WES sequencing of patients with ASD led to estimates that 500–1000 genes contribute to disease etiology [19], whereas in ID this number is greater than 1000 [20]. Epilepsy and schizophrenia are thought to be less genetically heterogeneous, involving approximately 500 [21] and 600 [22] genes, respectively. Although associations between certain gene variants and ND risk have been consistently replicated (such as de novo disruptive mutations in *CHD8*, *ADNP* and *DYRK1A* [19] among ASD and ID simplex families), hundreds of ND risk genes remain undiscovered or have not been associated with NDs with sufficient statistical significance owing to ultra-low mutation frequencies in the patient population.

**Table 2:** Genes linked to NDs identified through genotype-first approaches

Gene	Locus	Phenotype	References
*ADNP*	20q13.13	ASD, ID, loss of expressive language, facial dysmorphisms	[17]
*AHDC1*	1p35.3	ID/DD, hypotonia, mild dysmorphic facial features, sleep apnea	[59]
*ANKRD11*	16q24.3	KGB syndrome: macrodontia of central upper incisors, dental anomalies, facial dysmorphism, ASD, hyperactivity, hearing loss, heart defects	[60, 61]
*ARID1A*	1p36.11	Coffin–Siris syndrome with sucking/feeding difficulties, distinct faces including some facial coarseness with short nose, gastrointestinal complications, some hearing and visual impairments, prone to infection, hypotonia, structural CNS abnormalities, mild to severe ID/DD, behavioral abnormalities	[62, 63]
*ARID1B*	6q25.3	Highly variable Coffin–Siris syndrome with hypoplasia of the corpus callosum, hearing problems	[62, 64]
*ARID2*	12q12	ID/DD, ADHD, short stature, dysmorphic facial features, Wormian bones	[65]
*AUTS2*	7q11.22	ID, ASD, short stature, microcephaly, cerebral palsy, facial dysmorphism	[66]
*BRWD3*	Xq21.1	Mild to moderate ID, speech delay, behavioral disturbances, macrocephaly, dysmorphic facial features, skeletal symptoms	[67]
*CHAMP1*	13q34	ID, speech impairment, motor DD, muscular hypotonia, dysmorphic features	[68]
*CHD2*	15q26.1	Archetypal generalized photosensitive epilepsy syndrome, eyelid myoclonia with absences, ID, ± ASD	[38, 69–71]
*CHD8*	14q11.2	ASD, macrocephaly, distinct faces, gastrointestinal complaint, sleep disturbance	[16]
*CTNNB1*	3p22.1	ID, microcephaly, limited speech and progressive spasticity	[72, 73]
*CUL4B*	Xq24	X-linked ID, cerebral malformations, seizures, tremors, gait abnormalities, behavioral problems, macrocephaly, short stature, obesity, hypogonadotropic hypogonadism, variable dysmorphic features	[74]
*DDX3X*	Xp11.4	ID, hypotonia, movements disorders, behavior problems, corpus callosum hypoplasia, EP	[53]
*DEAF1*	11p15.5	Severe ID, severely affected speech development, behavioral problems	[75]
*DYNC1H1*	14q32.31	Severe ID, neuronal migration defects, broad phenotypic spectrum	[76]
*DYRK1A*	21q22.13	DD, ASD, microcephaly, late-onset EP, severe anxiety	[18]
*GATAD2B*	1q21.3	Severe ID, childhood hypotonia, limited speech, tubular nose with broad nasal tip, short philtrum, sparse hair and strabismus	[77]
*GRIN2A*	16p13.2	Epilepsy–aphasia spectrum disorders	[78]
*HDAC8*	Xq13.1	ID, behavioral problems (including ASD in some patients), delayed anterior fontanelle closure, ocular hypertelorism, hooding of the eyelids, broad nose and dental anomalies	[79]
*HIVEP2*	6q24.2	ID, structural brain anomalies, hypotonia, mild dysmorphic features	[80]
*KCNH1*	1q32.2	Severe ID, neonatal hypotonia, hypertelorism, broad nasal tip, wide mouth, nail aplasia or hypoplasia, proximal implanted and long thumb and long great toes	[81]
*KCNT1*	9q34.3	Highly pleiotropic, including nocturnal frontal lobe EP, malignant migrating focal seizures of infancy, Ohtahara syndrome, multifocal EP, cardiac disturbances	[82]
*KDM5C*	Xp11.22	X-linked ID	[83]
*KIAA2022*	Xq13.3	X-linked ID, ASD features, strabismus	[84]
*KIF1A*	2q37.3	Cognitive impairment, variable cerebellar atrophy, spastic paraparesis, optic nerve atrophy, peripheral neuropathy, EP	[85]
*KIF4A*	Xq13.1	ID, EP	[86]
*KIF5C*	2q23.1	Severe ID, EP, microcephaly, cortical malformation	[86]
*MAN1B1*	9q34.3	ID, delayed motor and speech development, obesity, macrocephaly	[87]
*MLL2 (KMT2D)*	12q13.12	Severe form of Kabuki syndrome with distinct facial features (large dysplastic ears, arched eyebrows with sparse lateral third, blue sclerae, flat nasal tip with a broad nasal root, a thin upper lip and a full lower lip)	[88]
*MYT1L*	2p25.3	Syndromic ID and/or central obesity	[89]
*NRXN1*	2p16.3	Predisposition for idiopathic generalized EP syndromes	[90]
*PGAP1*	2q33.1	ID, cerebral visual impairment	[91]
*PHF21A*	11p11.2	Potocki–Shaffer syndrome associated with ID and craniofacial anomalies	[92]
*PPP2R1A*	19q13.41	ID, hypotonia, EP, frontal bossing, mild hypertelorism, downslanting palpebral fissures	[93]
*PPP2R5D*	6p21.1	ID, ASD, macrocephaly, hypotonia, seizures, dysmorphic features	[93, 94]
*SCN1A*	2q24.3	EP with febrile seizures plus generalized epilepsy with febrile seizure plus (GEFS+), epilepsy–aphasia	[38]
*SCN2A*	2q24.3	ID/DD, seizure disorders (epileptic encephalopathy); previously implicated in ASD and SZ	[38, 95, 96]
*SCN8A*	12q13.13	Early-infantile epileptic encephalopathy type 13, ± ID, hypotonia, movement disorders	[38, 97, 98]
*SETBP1*	18q12.3	ID, loss of expression language	[99]
*SETD5*	3p25.3	ID, facial dysmorphism	[100]
*SLC6A1*	3p25.3	Spontaneous spike-wave discharges associated with epileptic encephalopathy	[101]
*SMARCA4*	19p13.2	Coffin–Siris syndrome with moderately coarse craniofacial features and behavioral abnormalities	[62, 63]
*SMARCB1*	22q11.23	Coffin–Siris syndrome with severe neurodevelopmental deficiencies, including severe ID, seizures, CNS structural abnormalities, no expressive words, scoliosis	[62, 63]
*SMARCE1*	17q21.2	Coffin–Siris syndrome with severe to moderate ID and a wide spectrum of manifestations	[63]
*SMC3*	10q25.2	Cornelia de Lange syndrome with postnatal microcephaly, moderately distinctive craniofacial appearance, mild prenatal growth retardation that worsens in childhood, some congenital heart defects, absence of limb deficiencies	[102]
*SRCAP*	16p11.2	Floating–Harbor syndrome: short stature with delayed bone age, deficits in expressive language, distinctive faces	[103]
*SYNGAP1*	6p21.32	Multiple seizure types, early DD, regression, ID	[38]
*USP9X*	Xp11.4	X-linked ID	[104]
*WAC*	10p11.23	ID/DD, hypotonia, behavioral problems, eye abnormalities, constipation, feeding difficulties, seizures, sleep problems, dysmorphic features	[105]
*ZMYND11*	10p15.3	ID, ASD, aggression, complex neuropsychiatric features	[99]

*ADHD* Attention deficit hyperactivity disorder, *ASD* Autism spectrum disorders, *CNS* Central nervous system, *DD* Developmental delay, *EP* Epilepsy, *ID* Intellectual disability, *SZ* Schizophrenia

We combined the results of multiple published WES, WGS and targeted sequencing studies including 5001–5922 individuals with ASD (single gene denominators varied owing to the variety of WES, WGS and targeted sequencing approaches used) [19, 23–28], 1284 individuals with ID [29–31], 274 individuals with epilepsy [32, 33] and 785 individuals with schizophrenia [34–36] to look for genetic overlap between these NDs. Using this large dataset (over 7000 individuals/families), we identified the top 25 genes that show an excess of disruptive (frameshift, splice, nonsense) gene mutations in disease cases when these individuals are compared with 45,376 controls drawn from the ExAC database [37], where neuropsychiatric cases were masked before analysis (Table 1). Although the number of individuals represented in each disease study differs and several genes reach only nominal significance, the identified genes clearly converge on common biochemical and neurodevelopmental pathways, such as synaptic function, chromatin remodeling, gene transcription and Wnt/β-catenin signaling. Importantly, significance thresholds are likely to be highly conservative, as the ND studies that were included in the analysis only considered confirmed de novo events, whereas the ExAC database variants have not been filtered for population frequency and inheritance status is unknown. Using our large dataset of de novo mutations associated with ND we can apply a recurrent de novo simulation model which considers the size and evolutionary conservation of individual genes to calculate the likelihood of observing a number of de novo mutations in any given ND-associated gene [23]. In some cases we find that genes that were not statistically significant for overall disruptive mutational burden after Bonferroni correction (*p* < 10-6) of the Fisher’s exact test *p* value are indeed significant for recurrent de novo mutation burden — such as *GRIN2B*, which has a de novo *p* value of 0.001 after correction. Therefore, although some genes (e.g., *GRIN2B*) reach only nominal significance for an overall increased burden in disruptive mutations in ND cases compared with unaffected controls, based on a de novo model they may prove to be bona fide ND risk genes.

The discovery of recurrently mutated genes has been used to successfully identify additional patients with disruptive mutations in these risk genes who, when collectively phenotyped, define new syndromic and sub-syndromic forms of ND [16–18]. These efforts have proceeded in parallel with the coordination of clinical exome sequencing of patients, which has led to the identification of dozens of individuals with the same type of rare molecular defect (Table 2). This coordination led to the emergence of refined patient checklists that enable a systematic reassessment of pediatric, neuroimaging, neurobehavioral and morphological features [15]. Such genotype–phenotype studies have shown that individuals sharing a genetic etiology have more features in common than those they share with the general ND population. These observations have led to the description of both genetic and clinical subtypes of ND, some of which may be considered syndromic by clinicians.

Many of the top ND risk genes identified in our analysis are correlated with an observable phenotype that may have been predicted based on our analysis (Tables 1 and 2). For example, *CHD8* is an ASD-associated gene linked with macrocephaly and gastrointestinal dysfunction [16], whereas *ADNP* mutations are associated with ASD and the complete loss of expressive language [17]. Some genes seem to be predominantly associated with ID (e.g., *ARID1B*, *ANKRD11*, *CTNNB1*, *STXBP1* and *CHAMP1*). *SCN1A* mutations have been primarily observed in epilepsy [38]. Other genes are strongly associated with epilepsy and ID (e.g., *CHD2* and *DYRK1A*), often with very specific clinical manifestations (e.g., microcephaly and late-onset epilepsy in the case of patients with *DYRK1A* variants [18]. The potential contribution of some of these ND genes (e.g., *SCN2A*, *CHD8* and *POGZ*; Table 1) to adult neuropsychiatric diseases, such as schizophrenia, is intriguing, although statistical significance supporting these associations is still lacking. The existence of such associations would suggest that mutations in these genes have broad phenotypic effects or variable expressivity that manifests as ND at different developmental stages. It will be important to identify families in which gene-disruptive mutations in these genes are segregated in order to explore phenotypic differences among the familial carriers.

## Molecular pathways and therapeutic potential

Beyond genetic subtypes, network-based approaches that more globally predict the effects of ND risk genes on molecular pathways have repeatedly shown an enrichment for synapse function and gene transcription/chromatin remodeling [19, 39]. Although these pathways remain the most statistically significant pathways found among ND datasets, other pathways have been identified, including interaction with SNARE proteins and vesicular transport pathways in epilepsy (*p* < 0.03) and FMRP targets in ASD, ID and epilepsy (*p* < 0.00001) [39]. Given the extensive locus heterogeneity of these diseases, pathway-defined ‘molecular subtypes’ are likely to become the ultimate target for behavioral and pharmacological therapeutics.

Each of these large functional networks can be further subdivided into smaller pathways, such as long-term potentiation, calcium signaling, postsynaptic density and synapse structure in the case of synaptic function, in which enrichment is driven by signals from de novo mutations in genes such as *SYNGAP1*, *SCN2A*, *STXBP1*, *GRIN2B* and *SCN1A* (Table 1). *SCN2A* and *SCN1A* are members of the same gene family of voltage-gated sodium channels that are responsible for the generation and propagation of action potentials and have been associated with seizure phenotypes in animal models [38]. Although SCN1A de novo mutations seem to be specific to epilepsy [38], we observe SCN2A de novo mutations in both ASD and ID (Table 1), which suggests that long-term potentiation has a role in multiple forms of ND. It is important to note, however, that we are classifying mutations using the primary clinical diagnosis under which each patient’s cohort was originally ascertained. As a large phenotypic overlap exists between NDs, we could reasonably hypothesize, for example, that patients with ASD or ID and an *SCN2A* mutation could also manifest with seizure phenotypes.

An enrichment for synapse function in ND has been observed primarily in a subset of patients with ID, epilepsy and schizophrenia [39]. Many antipsychotic and psychotropic compounds have been developed to modulate synaptic function to treat comorbid conditions (hyperactivity, depression, anxiety, aggression and seizures) often associated with NDs. These medications may be used more effectively when applied to patients with a molecular perturbation in the relevant gene or pathway. For example, benzodiazepines (e.g., clonazepam) are a class of drugs that increase GABA_A_ receptor activity and thus contribute to the inhibition of action potentials in the central nervous system, which are often overactive in seizure conditions [40]. Efforts are currently underway to specifically tailor benzodiazepines to treat patients with mutations in *SCN2A* and *SCN1A* [41, 42] (Dr. Raphael Bernier, personal communication). Clemizole, a compound approved by the US Food and Drug Administration, has been shown to mitigate some of the convulsive behavior of *Scn1a* mutant zebrafish [43]. *Scn2a* mutant mice are being used in the development of other similar sodium-channel-inhibiting compounds, including GS967 [44].

Studies of simplex ASD and ID families have highlighted an enrichment for gene-disruptive mutations in transcription and chromatin remodeling pathways (e.g., SWI/SNF complex, Wnt/β-catenin and mTOR) [19, 39, 45]. Wnt/β-catenin and mTOR pathways are involved in gene transcription, cell growth, migration and patterning during embryonic development [46, 47]. These pathways are closely linked to the SWI/SNF nucleosome remodeling complex, which is involved in the regulation of gene expression and is thought to have a role in neural specification [48]. Understanding the molecular biology of these pathways may reveal additional therapeutic targets. *ADNP*, for example, is a transcription factor that interacts directly with the SWI/SNF complex. Davunetide, a derivative octapeptide of *ADNP*, has been shown to ameliorate some of the cognitive deficits in animal models with *ADNP* mutations, which is a promising line of therapeutic research for ADNP patients with similar defects [49]. Some ND-associated genes (Table 1) are simultaneously involved in chromatin remodeling and transcription, such as *ARID1B* [50] and *CHD8* [51], which have been linked to the SWI/SNF and Wnt/β-catenin signaling pathways [16, 50] (Table 1) and are known to be important for proliferation of neural precursors [23, 39, 52].The study of genetic subtypes of ND associated with the Wnt/β-catenin pathway — specifically *DDX3X* and *CHD8* — suggests that mutations in this pathway are important in the very early stages of development [16, 53]. Importantly, mutations in *DDX3X* account for a large percentage of unexplained ID in female individuals (1–3 %) [53], which was overlooked in studies of ASD alone [54] (Table 1). The Wnt/β-catenin pathway is commonly dysregulated in cancer; over 40 compounds have been shown to modulate Wnt/β-catenin pathway activity in model systems or in vivo that might be considered for use in specific genetic subtypes of ND in the future [55].

Mutations in the mTOR pathway involving genes such as *TSC* and *PTEN* have also been implicated in tumorigenesis and ND owing to their role in transcription and cell growth [47]. Rapalogues, including sirolimus (rapamycin) and everolimus, which inhibit TORC1 and are commonly used to treat cancer, are currently under investigation to assess whether they can improve ASD-related symptoms in patients with *TSC* mutations [56]. Similar disease-modifying therapies might be useful to treat patients with other genetic subtypes of ND in which mTOR function is abrogated. However, the use of drugs targeting both Wnt/β-catenin and mTOR pathways will need to be carefully considered and fine-tuned for use in NDs to avoid adverse side effects. Although killing healthy cells in adults is an acceptable consequence of cancer treatment, this is not the case during pediatric brain development.

## Conclusions

The success of the genotype-first approach for subtyping NDs can be primarily attributed to technological advances that make WES and targeted sequencing fast and cost-effective. ND candidate gene discovery can be maximized by combining many datasets from overlapping conditions (e.g., ASD, ID, epilepsy and schizophrenia) to (1) increase the genetic evidence supporting individual ND risk gene models, (2) build stronger molecular interaction networks that implicate specific pathways in disease biology and (3) assess the robustness of genotype–phenotype links. Beyond providing a potential genetic explanation for disease to families, our understanding of the biological pathways that are disrupted by specific variants is leading to improved assessment of disease risk in families and to the prospect of tailored treatments for patients with these debilitating diseases.

## Abbreviations

ASD: Autism spectrum disorders
DSM: Diagnostic and Statistical Manual of Mental Disorders
ID: Intellectual disability
ND: Neurodevelopmental disorder
WES: Whole-exome sequencing
WGS: Whole-genome sequencing

## References

[1] Carreno M, Donaire A, Sanchez-Carpintero R (2008). Cognitive disorders associated with epilepsy: diagnosis and treatment. Neurologist.

[2] American Psychiatric Association. Diagnostic and statistical manual of mental disorders. 5th ed. New York: American Psychiatric Association; 2013.

[3] McPartland JC, Reichow B, Volkmar FR (2012). Sensitivity and specificity of proposed DSM-5 diagnostic criteria for autism spectrum disorder. J Am Acad Child Adolesc Psychiatry.

[4] Hallmayer J, Cleveland S, Torres A, Phillips J, Cohen B, Torigoe T (2011). Genetic heritability and shared environmental factors among twin pairs with autism. Arch Gen Psychiatry.

[5] Bailey A, Le Couteur A, Gottesman I, Bolton P, Simonoff E, Yuzda E (1995). Autism as a strongly genetic disorder: evidence from a British twin study. Psychol Med.

[6] Steffenburg S, Gillberg C, Hellgren L, Andersson L, Gillberg IC, Jakobsson G (1989). A twin study of autism in Denmark, Finland, Iceland, Norway and Sweden. J Child Psychol Psychiatry.

[7] Kjeldsen MJ, Kyvik KO, Christensen K, Friis ML (2001). Genetic and environmental factors in epilepsy: a population-based study of 11900 Danish twin pairs. Epilepsy Res.

[8] Lichtenstein P, Yip BH, Bjork C, Pawitan Y, Cannon TD, Sullivan PF (2009). Common genetic determinants of schizophrenia and bipolar disorder in Swedish families: a population-based study. Lancet.

[9] Sullivan PF, Kendler KS, Neale MC (2003). Schizophrenia as a complex trait: evidence from a meta-analysis of twin studies. Arch Gen Psychiatry.

[10] Bryson SE, Bradley EA, Thompson A, Wainwright A (2008). Prevalence of autism among adolescents with intellectual disabilities. Can J Psychiatry.

[11] McGrother CW, Bhaumik S, Thorp CF, Hauck A, Branford D, Watson JM (2006). Epilepsy in adults with intellectual disabilities: prevalence, associations and service implications. Seizure.

[12] Morgan VA, Leonard H, Bourke J, Jablensky A (2008). Intellectual disability co-occurring with schizophrenia and other psychiatric illness: population-based study. Br J Psychiatry.

[13] Moreno-De-Luca A, Myers SM, Challman TD, Moreno-De-Luca D, Evans DW, Ledbetter DH (2013). Developmental brain dysfunction: revival and expansion of old concepts based on new genetic evidence. Lancet Neurol.

[14] Torres F, Barbosa M, Maciel P (2015). Recurrent copy number variations as risk factors for neurodevelopmental disorders: critical overview and analysis of clinical implications. J Med Genet.

[15] Stessman HA, Bernier R, Eichler EE (2014). A genotype-first approach to defining the subtypes of a complex disease. Cell.

[16] Bernier R, Golzio C, Xiong B, Stessman HA, Coe BP, Penn O (2014). Disruptive CHD8 mutations define a subtype of autism early in development. Cell.

[17] Helsmoortel C, Vulto-van Silfhout AT, Coe BP, Vandeweyer G, Rooms L, van den Ende J (2014). A SWI/SNF-related autism syndrome caused by de novo mutations in ADNP. Nat Genet.

[18] van Bon BW, Coe BP, Bernier R, Green C, Gerdts J, Witherspoon K (2015). Disruptive de novo mutations of DYRK1A lead to a syndromic form of autism and ID. Mol Psychiatry.

[19] Sanders SJ, He X, Willsey AJ, Ercan-Sencicek AG, Samocha KE, Cicek AE (2015). Insights into autism spectrum disorder genomic architecture and biology from 71 risk loci. Neuron.

[20] van Bokhoven H (2011). Genetic and epigenetic networks in intellectual disabilities. Annu Rev Genet.

[21] Noebels J (2015). Pathway-driven discovery of epilepsy genes. Nat Neurosci.

[22] Schizophrenia Working Group of the Psychiatric Genomics Consortium. Biological insights from 108 schizophrenia-associated genetic loci. Nature. 2014;511(7510):421–7. doi:10.1038/nature13595. 10.1038/nature13595 PMC4112379 25056061

[23] O'Roak BJ, Vives L, Fu W, Egertson JD, Stanaway IB, Phelps IG (2012). Multiplex targeted sequencing identifies recurrently mutated genes in autism spectrum disorders. Science.

[24] O'Roak BJ, Stessman HA, Boyle EA, Witherspoon KT, Martin B, Lee C (2014). Recurrent de novo mutations implicate novel genes underlying simplex autism risk. Nat Commun.

[25] Michaelson JJ, Shi Y, Gujral M, Zheng H, Malhotra D, Jin X (2012). Whole-genome sequencing in autism identifies hot spots for de novo germline mutation. Cell.

[26] Jiang YH, Yuen RK, Jin X, Wang M, Chen N, Wu X (2013). Detection of clinically relevant genetic variants in autism spectrum disorder by whole-genome sequencing. Am J Hum Genet.

[27] Tavassoli T, Kolevzon A, Wang AT, Curchack-Lichtin J, Halpern D, Schwartz L (2014). De novo SCN2A splice site mutation in a boy with Autism spectrum disorder. BMC Med Genet.

[28] Yuen RK, Thiruvahindrapuram B, Merico D, Walker S, Tammimies K, Hoang N (2015). Whole-genome sequencing of quartet families with autism spectrum disorder. Nat Med.

[29] de Ligt J, Willemsen MH, van Bon BW, Kleefstra T, Yntema HG, Kroes T (2012). Diagnostic exome sequencing in persons with severe intellectual disability. N Engl J Med.

[30] Rauch A, Wieczorek D, Graf E, Wieland T, Endele S, Schwarzmayr T (2012). Range of genetic mutations associated with severe non-syndromic sporadic intellectual disability: an exome sequencing study. Lancet.

[31] Deciphering Developmental Disorders Study (2015). Large-scale discovery of novel genetic causes of developmental disorders. Nature.

[32] Epi4K Consortium; Epilepsy Phenome/Genome Project, Allen AS, Berkovic SF, Cossette P, Delanty N, et al. De novo mutations in epileptic encephalopathies. Nature. 2013;501(7466):217–21. doi:10.1038/nature12439. 10.1038/nature12439 PMC3773011 23934111

[33] Veeramah KR, Johnstone L, Karafet TM, Wolf D, Sprissler R, Salogiannis J (2013). Exome sequencing reveals new causal mutations in children with epileptic encephalopathies. Epilepsia.

[34] Gulsuner S, Walsh T, Watts AC, Lee MK, Thornton AM, Casadei S (2013). Spatial and temporal mapping of de novo mutations in schizophrenia to a fetal prefrontal cortical network. Cell.

[35] McCarthy SE, Gillis J, Kramer M, Lihm J, Yoon S, Berstein Y (2014). De novo mutations in schizophrenia implicate chromatin remodeling and support a genetic overlap with autism and intellectual disability. Mol Psychiatry.

[36] Fromer M, Pocklington AJ, Kavanagh DH, Williams HJ, Dwyer S, Gormley P (2014). De novo mutations in schizophrenia implicate synaptic networks. Nature.

[37] Consortium EA. ExAC Browser. Cambridge, MA. 2015. http://exac.broadinstitute.org/. Accessed 8 September 2015.

[38] Carvill GL, Heavin SB, Yendle SC, McMahon JM, O'Roak BJ, Cook J (2013). Targeted resequencing in epileptic encephalopathies identifies de novo mutations in CHD2 and SYNGAP1. Nat Genet.

[39] Hormozdiari F, Penn O, Borenstein E, Eichler EE (2015). The discovery of integrated gene networks for autism and related disorders. Genome Res.

[40] Johnston GA (1996). GABAA receptor pharmacology. Pharmacol Therapeut.

[41] Han S, Tai C, Westenbroek RE, Yu FH, Cheah CS, Potter GB (2012). Autistic-like behaviour in Scn1a+/- mice and rescue by enhanced GABA-mediated neurotransmission. Nature.

[42] Han S, Tai C, Jones CJ, Scheuer T, Catterall WA (2014). Enhancement of inhibitory neurotransmission by GABAA receptors having alpha2,3-subunits ameliorates behavioral deficits in a mouse model of autism. Neuron.

[43] Baraban SC, Dinday MT, Hortopan GA (2013). Drug screening in Scn1a zebrafish mutant identifies clemizole as a potential Dravet syndrome treatment. Nat Commun.

[44] Anderson LL, Thompson CH, Hawkins NA, Nath RD, Petersohn AA, Rajamani S (2014). Antiepileptic activity of preferential inhibitors of persistent sodium current. Epilepsia.

[45] O'Roak BJ, Vives L, Girirajan S, Karakoc E, Krumm N, Coe BP (2012). Sporadic autism exomes reveal a highly interconnected protein network of de novo mutations. Nature.

[46] Patapoutian A, Reichardt LF (2000). Roles of Wnt proteins in neural development and maintenance. Curr Opin Neurobiol.

[47] Jaworski J, Sheng M (2006). The growing role of mTOR in neuronal development and plasticity. Mol Neurobiol.

[48] Battaglioli E, Andres ME, Rose DW, Chenoweth JG, Rosenfeld MG, Anderson ME (2002). REST repression of neuronal genes requires components of the hSWI.SNF complex. J Biol Chem.

[49] Vandeweyer G, Helsmoortel C, Van Dijck A, Vulto-van Silfhout AT, Coe BP, Bernier R (2014). The transcriptional regulator ADNP links the BAF (SWI/SNF) complexes with autism. Am J Med Genet C Semin Med Genet.

[50] Vasileiou G, Ekici AB, Uebe S, Zweier C, Hoyer J, Engels H (2015). Chromatin-Remodeling-Factor ARID1B Represses Wnt/beta-Catenin Signaling. Am J Hum Genet.

[51] Nishiyama M, Skoultchi AI, Nakayama KI (2012). Histone H1 recruitment by CHD8 is essential for suppression of the Wnt-beta-catenin signaling pathway. Mol Cell Biol.

[52] Hirabayashi Y, Itoh Y, Tabata H, Nakajima K, Akiyama T, Masuyama N (2004). The Wnt/beta-catenin pathway directs neuronal differentiation of cortical neural precursor cells. Development.

[53] Snijders Blok L, Madsen E, Juusola J, Gilissen C, Baralle D, Reijnders MR (2015). Mutations in DDX3X are a common cause of unexplained intellectual disability with gender-specific effects on Wnt signaling. Am J Hum Genet.

[54] Iossifov I, O'Roak BJ, Sanders SJ, Ronemus M, Krumm N, Levy D (2014). The contribution of de novo coding mutations to autism spectrum disorder. Nature.

[55] Anastas JN, Moon RT (2013). WNT signalling pathways as therapeutic targets in cancer. Nat Rev Cancer.

[56] Rapalogues for Autism Phenotype in TSC: a feasibility study (RAPT). https://clinicaltrials.gov/ct2/show/NCT01929642?term=NCT01929642&rank=1. 2013. Accessed 1 November 2015.

[57] Huang DW, Sherman BT, Lempicki RA (2009). Bioinformatics enrichment tools: paths toward the comprehensive functional analysis of large gene lists. Nucleic Acids Res.

[58] Huang DW, Sherman BT, Lempicki RA (2009). Systematic and integrative analysis of large gene lists using DAVID bioinformatics resources. Nat Protoc.

[59] Xia F, Bainbridge MN, Tan TY, Wangler MF, Scheuerle AE, Zackai EH (2014). De novo truncating mutations in AHDC1 in individuals with syndromic expressive language delay, hypotonia, and sleep apnea. Am J Hum Genet.

[60] Ockeloen CW, Willemsen MH, de Munnik S, van Bon BWM, de Leeuw N, Verrips A (2015). Further delineation of the KBG syndrome phenotype caused by ANKRD11 aberrations. Eur J Hum Genet.

[61] Isrie M, Hendriks Y, Gielissen N, Sistermans EA, Willemsen MH, Peeters H (2012). Haploinsufficiency of ANKRD11 causes mild cognitive impairment, short stature and minor dysmorphisms. Eur J Hum Genet.

[62] Santen GWE, Aten E, Vulto-van Silfhout AT, Pottinger C, van Bon BWM, van Minderhout IJHM (2013). Coffin-Siris syndrome and the BAF complex: genotype-phenotype study in 63 patients. Hum Mutat.

[63] Kosho T, Okamoto N, Collaborators C-SSI (2014). Genotype-phenotype correlation of Coffin-Siris syndrome caused by mutations in SMARCB1, SMARCA4, SMARCE1, and ARID1A. Am J Med Genet C Semin Med Genet.

[64] Santen GWE, Clayton-Smith J; ARID1B-CSS consortium. The ARID1B phenotype: what we have learned so far. Am J Med Genet C Semin Med Genet. 2014;166C:276–89. doi:10.1002/ajmg.c.31414. 10.1002/ajmg.c.31414 25169814

[65] Shang L, Cho MT, Retterer K, Folk L, Humberson J, Rohena L (2015). Mutations in ARID2 are associated with intellectual disabilities. Neurogenetics.

[66] Beunders G, Voorhoeve E, Golzio C, Pardo LM, Rosenfeld JA, Talkowski ME (2013). Exonic deletions in AUTS2 cause a syndromic form of intellectual disability and suggest a critical role for the C terminus. Am J Hum Genet.

[67] Grotto S, Drouin-Garraud V, Ounap K, Puusepp-Benazzouz H, Schuurs-Hoeijmakers J, Le Meur N, et al. Clinical assessment of five patients with BRWD3 mutation at Xq21.1 gives further evidence for mild to moderate intellectual disability and macrocephaly. Eur J Med Genet. 2014;57:200–6. doi:10.1016/j.ejmg.2013.12.012. 10.1016/j.ejmg.2013.12.012 24462886

[68] Hempel M, Cremer K, Ockeloen CW, Lichtenbelt KD, Herkert JC, Denecke J (2015). De novo mutations in CHAMP1 cause intellectual disability with severe speech impairment. Am J Hum Genet.

[69] Chenier S, Yoon G, Argiropoulos B, Lauzon J, Laframboise R, Ahn JW (2014). CHD2 haploinsufficiency is associated with developmental delay, intellectual disability, epilepsy and neurobehavioural problems. J Neurodev Disord.

[70] Thomas RH, Zhang LM, Carvill GL, Archer JS, Heavin SB, Mandelstam SA (2015). CHD2 myoclonic encephalopathy is frequently associated with self-induced seizures. Neurology.

[71] Galizia EC, Myers CT, Leu C, de Kovel CGF, Afrikanova T, Cordero-Maldonado ML (2015). CHD2 variants are a risk factor for photosensitivity in epilepsy. Brain.

[72] Kuechler A, Willemsen MH, Albrecht B, Bacino CA, Bartholomew DW, van Bokhoven H (2015). De novo mutations in beta-catenin (CTNNB1) appear to be a frequent cause of intellectual disability: expanding the mutational and clinical spectrum. Hum Genet.

[73] Tucci V, Kleefstra T, Hardy A, Heise I, Maggi S, Willemsen MH (2014). Dominant β-catenin mutations cause intellectual disability with recognizable syndromic features. J Clin Invest.

[74] Vulto-van Silfhout AT, Nakagawa T, Bahi-Buisson N, Haas SA, Hu H, Bienek M (2015). Variants in CUL4B are associated with cerebral malformations. Hum Mutat.

[75] Vulto-van Silfhout AT, Rajamanickam S, Jensik PJ, Vergult S, de Rocker N, Newhall KJ (2014). Mutations affecting the SAND domain of DEAF1 cause intellectual disability with severe speech impairment and behavioral problems. Am J Hum Genet.

[76] Willemsen MH, Vissers LEL, Willemsen MAAP, van Bon BWM, Kroes T, de Ligt J (2012). Mutations in DYNC1H1 cause severe intellectual disability with neuronal migration defects. J Med Genet.

[77] Willemsen MH, Nijhof B, Fenckova M, Nillesen WM, Bongers EM, Castells-Nobau A (2013). GATAD2B loss-of-function mutations cause a recognisable syndrome with intellectual disability and are associated with learning deficits and synaptic undergrowth in Drosophila. J Med Genet.

[78] Carvill GL, Regan BM, Yendle SC, O'Roak BJ, Lozovaya N, Bruneau N (2013). GRIN2A mutations cause epilepsy-aphasia spectrum disorders. Nat Genet.

[79] Kaiser FJ, Ansari M, Braunholz D, Concepción Gil-Rodríguez M, Decroos C, Wilde JJ (2014). Loss-of-function HDAC8 mutations cause a phenotypic spectrum of Cornelia de Lange syndrome-like features, ocular hypertelorism, large fontanelle and X-linked inheritance. Hum Mol Genet.

[80] Srivastava S, Engels H, Schanze I, Cremer K, Wieland T, Menzel M (2015). Loss-of-function variants in HIVEP2 are a cause of intellectual disability. Eur J Hum Genet.

[81] Bramswig NC, Ockeloen CW, Czeschik JC, van Essen AJ, Pfundt R, Smeitink J (2015). 'Splitting versus lumping': Temple-Baraitser and Zimmermann-Laband asyndromes. Hum Genet.

[82] Møller RS, Heron SE, Larsen LHG, Lim CX, Ricos MG, Bayly MA (2015). Mutations in KCNT1 cause a spectrum of focal epilepsies. Epilepsia.

[83] Gonçalves TF, Gonçalves AP, Fintelman Rodrigues N, dos Santos JM, Pimentel MMG, Santos-Rebouças CB (2014). KDM5C mutational screening among males with intellectual disability suggestive of X-Linked inheritance and review of the literature. Eur J Med Genet.

[84] Van Maldergem L, Hou Q, Kalscheuer VM, Rio M, Doco-Fenzy M, Medeira A (2013). Loss of function of KIAA2022 causes mild to severe intellectual disability with an autism spectrum disorder and impairs neurite outgrowth. Hum Mol Genet.

[85] Lee J-R, Srour M, Kim D, Hamdan FF, Lim S-H, Brunel-Guitton C (2015). De novo mutations in the motor domain of KIF1A cause cognitive impairment, spastic paraparesis, axonal neuropathy, and cerebellar atrophy. Hum Mutat.

[86] Willemsen MH, Ba W, Wissink-Lindhout WM, de Brouwer APM, Haas SA, Bienek M (2014). Involvement of the kinesin family members KIF4A and KIF5C in intellectual disability and synaptic function. J Med Genet.

[87] Van Scherpenzeel M, Timal S, Rymen D, Hoischen A, Wuhrer M, Hipgrave-Ederveen A (2014). Diagnostic serum glycosylation profile in patients with intellectual disability as a result of MAN1B1 deficiency. Brain.

[88] Makrythanasis P, van Bon BW, Steehouwer M, Rodríguez-Santiago B, Simpson M, Dias P (2013). MLL2 mutation detection in 86 patients with Kabuki syndrome: a genotype-phenotype study. Clin Genet.

[89] De Rocker N, Vergult S, Koolen D, Jacobs E, Hoischen A, Zeesman S (2015). Refinement of the critical 2p25.3 deletion region: the role of MYT1L in intellectual disability and obesity. Genet Med.

[90] Møller RS, Weber YG, Klitten LL, Trucks H, Muhle H, Kunz WS (2013). Exon-disrupting deletions of NRXN1 in idiopathic generalized epilepsy. Epilepsia.

[91] Bosch DGM, Boonstra FN, Kinoshita T, Jhangiani S, de Ligt J, Cremers FPM (2015). Cerebral visual impairment and intellectual disability caused by PGAP1 variants. Eur J Hum Genet.

[92] Kim H-G, Kim H-T, Leach NT, Lan F, Ullmann R, Silahtaroglu A (2012). Translocations disrupting PHF21A in the Potocki-Shaffer-syndrome region are associated with intellectual disability and craniofacial anomalies. Am J Hum Genet.

[93] Houge G, Haesen D, Vissers LELM, Mehta S, Parker MJ, Wright M (2015). B56δ-related protein phosphatase 2A dysfunction identified in patients with intellectual disability. J Clin Invest.

[94] Shang L, Henderson LB, Cho MT, Petrey DS, Fong C-T, Haude KM (2015). De novo missense variants in PPP2R5D are associated with intellectual disability, macrocephaly, hypotonia, and autism. Neurogenetics.

[95] Carroll LS, Woolf R, Ibrahim Y, Williams HJ, Dwyer S, Walters J (2015). Mutation screening of SCN2A in schizophrenia and identification of a novel loss-of-function mutation. Psychiatric Genet.

[96] Howell KB, McMahon JM, Carvill GL, Tambunan D, Mackay MT, Rodriguez-Casero V (2015). SCN2A encephalopathy: a major cause of epilepsy of infancy with migrating focal seizures. Neurology.

[97] Blanchard MG, Willemsen MH, Walker JB, Dib-Hajj SD, Waxman SG, Jongmans MCJ (2015). De novo gain-of-function and loss-of-function mutations of SCN8A in patients with intellectual disabilities and epilepsy. J Med Genet.

[98] Larsen J, Carvill GL, Gardella E, Kluger G, Schmiedel G, Barisic N (2015). The phenotypic spectrum of SCN8A encephalopathy. Neurology.

[99] Coe BP, Witherspoon K, Rosenfeld JA, van Bon BW, Vulto-van Silfhout AT, Bosco P (2014). Refining analyses of copy number variation identifies specific genes associated with developmental delay. Nat Genet.

[100] Kuechler A, Zink AM, Wieland T, Lüdecke H-J, Cremer K, Salviati L (2015). Loss-of-function variants of SETD5 cause intellectual disability and the core phenotype of microdeletion 3p25.3 syndrome. Eur J Hum Genet.

[101] Carvill GL, McMahon JM, Schneider A, Zemel M, Myers CT, Saykally J (2015). Mutations in the GABA transporter SLC6A1 cause epilepsy with myoclonic-atonic seizures. Am J Hum Genet.

[102] Gil-Rodríguez MC, Deardorff MA, Ansari M, Tan CA, Parenti I, Baquero-Montoya C (2015). De novo heterozygous mutations in SMC3 cause a range of Cornelia de Lange syndrome-overlapping phenotypes. Hum Mutat.

[103] Nikkel SM, Dauber A, de Munnik S, Connolly M, Hood RL, Caluseriu O (2013). The phenotype of Floating-Harbor syndrome: clinical characterization of 52 individuals with mutations in exon 34 of SRCAP. Orphanet J Rare Dis.

[104] Homan CC, Kumar R, Nguyen LS, Haan E, Raymond FL, Abidi F (2014). Mutations in USP9X are associated with X-linked intellectual disability and disrupt neuronal cell migration and growth. Am J Hum Genet.

[105] DeSanto C, D'Aco K, Araujo GC, Shannon N, Study D, Vernon H, et al. WAC loss-of-function mutations cause a recognisable syndrome characterised by dysmorphic features, developmental delay and hypotonia and recapitulate 10p11.23 microdeletion syndrome. J Med Genet. 2015. doi:10.1136/jmedgenet-2015-103069. 10.1136/jmedgenet-2015-103069 26264232

